# Strategies of protected area use by Asian elephants in relation to motivational state and social affiliations

**DOI:** 10.1038/s41598-022-22989-1

**Published:** 2022-11-02

**Authors:** Anastasia E. Madsen, Christin Minge, T. V. Pushpakumara, U. Sameera Weerathunga, U. K. Padmalal, Devaka K. Weerakoon, Shermin de Silva

**Affiliations:** 1 University of Nebraska-Lincoln, Lincoln, NE USA; 2 Trunks and Leaves Inc, Newtonville, MA USA; 3 Institute of Ecology and Evolution, Friedrich-Schiller University, Jena, Germany; 4 EFECT, 215 A 3/7 Park Road, Colombo 5, Sri Lanka; 5 Open University of Sri Lanka, Colombo, Sri Lanka; 6 University of Colombo, Colombo, Sri Lanka; 7 University of California, San Diego, La Jolla, CA 92093 USA

**Keywords:** Behavioural ecology, Conservation biology

## Abstract

Animals’ space requirements may vary according to life-history and social considerations. We observed 516 wild adult Asian elephants from both sexes, over 9 years, to investigate how life-history traits and social behavior influence protected-area (PA) use at Udawalawe National Park, Sri Lanka. Male PA-use, quantified in terms of average between-sightings-interval (BSI), was significantly influenced by the interaction of age class and motivational state (i.e. reproduction vs. foraging). Musth lengthened with age, with a median of 24.5 days for ages 21–30, 32.5 days for ages 31–40, and 45 days for those > 40. A minority (11%) used it exclusively during musth, while others used it exclusively for foraging (44%) or both (45%). Males using it in both states and older musth-only males were more likely to be seen across years. There were 16 social communities containing between 2–22 adult females. Females’ BSI was significantly influenced by social ties, but this relationship was weak, because members of social communities do not necessarily disperse together, resulting in high individual variation in space-use. Inter-annual variability in sightings among individuals of both sexes indicates that around ¾ of the population is likely non-residential across years, challenging the prevailing fortress-conservation paradigm of wildlife management.

## Introduction

Global conservation goals typically emphasize setting aside land for wildlife^1^. Protected areas (PAs) can serve as refuges from anthropogenic impacts, both direct (e.g., harvest, hunting) and indirect (e.g., habitat modification). While much research has been devoted to developing metrics for prioritizing areas to protect on the basis of biodiversity or ecosystem services^2,3^, there has been relatively less emphasis on species’ behavior^4^. Indeed, some PAs may not meet conservation needs for certain taxa^5,6^, because globally PAs exhibit selection biases toward areas of low agricultural value rather than inherent ecological value^7^. Animal populations within PAs are also impacted by habitat degradation and activities outside PAs, as many species range beyond reserves^8^. Despite offering important cultural and economic benefits, dedicated reserves may not suffice to protect wildlife^8–11^.

Animal movements integrate PAs with wider landscapes^12^. Factors such as age, sex, and social status impact space use, interact with other life history traits, and can differ across the annual cycle. What fraction of any given population of threatened species actually relies on the PAs? How does PA-use relate to life history? Such considerations are as important as species or ecosystem diversity when designating and managing PAs^4,13^. We examine the PA-use strategies of a widely-distributed ecosystem engineer, the Asian elephant (*Elephas maximus*). Elephants move seeds, soil, and nutrients, influencing community structure^14,15^. Space use varies among species, habitats, and regions^16,17^, as well as between life history stages and throughout the annual cycle^18^. PAs in Asia tend to be small, with 80% of those in South Asia^19^ being < 100 km^2^, but they can be very important for wildlife when managed in conjunction with surrounding landscapes^20,21^. Unfortunately, they often have low connectivity^22,23^. Changing land-uses within and adjacent to elephant habitat alters the availability of both resources and cover, in turn affecting elephant space use, which increases potential for negative interactions as cropland and human settlements encroach^24–26^. These dynamics moreover erode the ecosystem services these species provide^27^. Yet, despite evident mismatches between the scale of elephants’ habitat requirements and that of PAs in Asia^28,29^, and concerns regarding their relationship to local communities^3,30^, the role of sanctuaries continue to be emphasized in both policy and practice^1,3^.

How elephants actually use PAs in relation to life-history remains poorly appreciated. Being a polygynandrous species with a slow reproductive rate, adults of opposite sex have distinct needs and do not consistently associate with one another^31^. Mature males alternate between periods prioritizing foraging vs. periods engaged in mate-search/competition, which we refer to as *foraging* or *reproductive motivational state*, respectively. The latter is known as “musth”^32^. This state of heightened sexual activity, which is highly asynchronous and individually-variable, is accompanied by a reduction in feeding, altered hormone profile, increased movement, and changes in associations with conspecifics^31,33–39^. Given these changes, the degree to which males use PAs in either motivational state is unclear. Adult female Asian elephants, on the other hand, exhibit fission–fusion social relationships, wherein social groups break apart and merge together across time and space^40,41^. Social relationships persist across years, even if individuals are not associated day-to-day, but lack the cohesiveness and seasonal stratification of African savannah elephants^42,43^. Their ability to move is thought to be an important mechanism for avoiding direct competition and conflicts, as dominance hierarchies appear largely absent^44^. Although social partners by definition have to be spatially associated at least some of the time, it is unknown whether affiliates in fact use the same areas as one another in the long-term.

**Figure 1:**
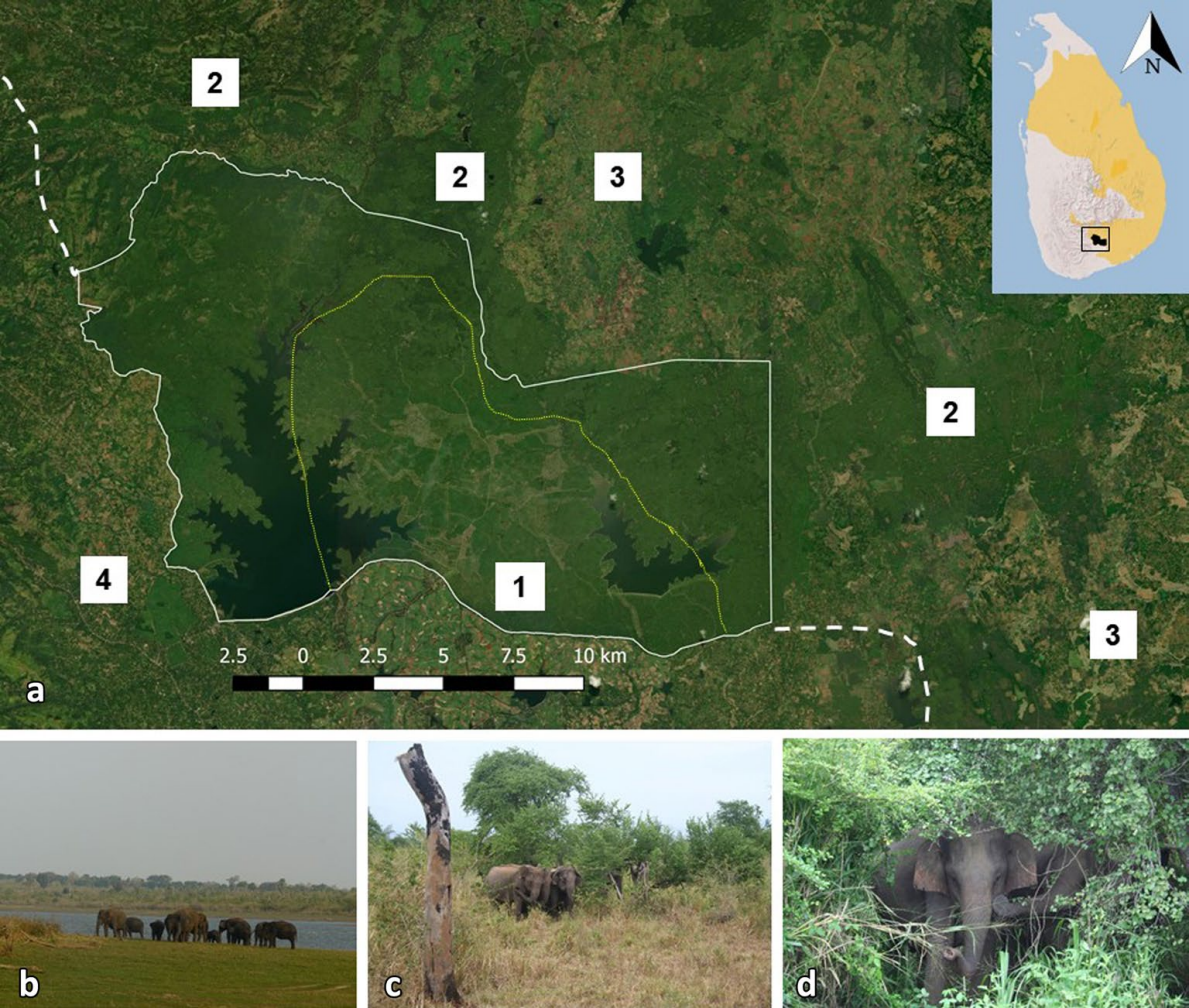
Study site. (**a**) Udawalawe National Park is located in southern Sri Lanka along one of the edges of elephant range in the country (shaded yellow, inset and white dashed line) and contains two reservoirs constructed between 1970 and 2000 as well as smaller water sources. Solid outlines show recognized park boundaries, dashed outlines show edge of elephant range. Area 1 (yellow dotted line) is the observation area with an accessible road network (visible); areas of type 2 represent forested sanctuaries or mixed-use lands of varying designations for which boundaries are not always clear; areas of type 3 represent agriculture and settlement mosaics in which elephants are present; areas of type 4 represent agricultural mosaics with denser settlements where elephants are absent except for occasional incursions by bulls. Areas 2–4 are shown for context, but observations were only performed in area 1. Basemap source: ESRI^®^ World Satellite Map. (**b**–**d**) Examples of habitat found in the study area, ranging from open floodplain to dense scrub.

While studies tracking the movements of individual animals provide perspective on home range attributes, they are typically limited in terms of the fraction of the population sampled and the duration of study. Using longitudinal data from direct observations of wild Asian elephants at Udawalawe National Park, Sri Lanka (Fig. 1), we investigate PA-use according to different considerations for the two sexes. For adult males, we examine whether individuals preferentially use the PA for foraging vs. mate search, hypothesizing that area use would be structured by age and motivational state. For adult females, we hypothesized that PA-use would be influenced by the strength of associations among social affiliates. We first quantify the degree to which elephants revisit the PA over multiple years, then evaluate the possible influences on area use strategies for each sex. We discuss how these findings complement recent studies from other parts of Asia and their implications for the function of PAs for this and other species.

## Results

### Male PA-use

**Figure 2:**
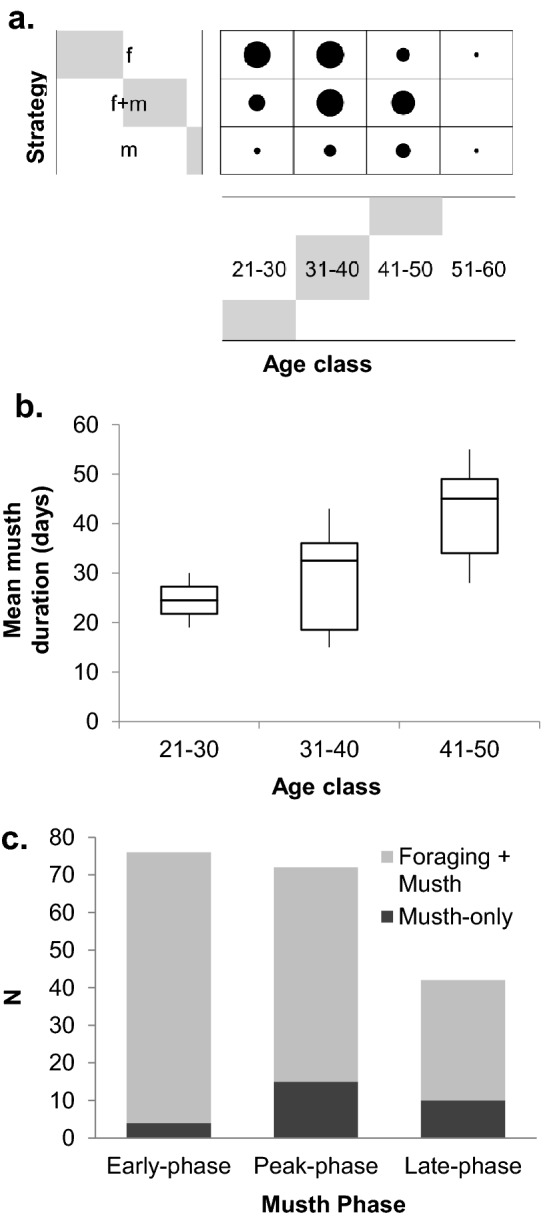
Musth expression and strategies. (**a**) Distribution of male ages across strategies (n = 216). The dot size reflects the relative number of males across age classes and musth categories. Grey bars are proportional to the total number of individuals observed in each row or column. Age class and strategy were significantly associated (Fisher’s exact test p < 0.0001). (**b**) Musth duration by age class. (**c**) Observed musth stages for males that were seen only in musth (n = 23), or those seen in both musth and foraging states (n = 99). More males are seen in the early phase than the peak or late phases, with the latter phases exhibited by more musth-only males.

We identified 379 individual males during the 6-year study period (Fig. S1), with those seen in musth distributed across age classes and overall number of sightings (Fig. S2). Detailed descriptions of the observation and analysis protocols are provided in the “Methods” (see also^40,45^). Of these, 216 were already mature (≥ 21 years old), whereas 25 that transitioned into maturity during the study and were excluded from analyses. Of the 216 mature males, 99 (45%) were seen only foraging, 94 (44%) were seen in both motivational states and 23 (11%) were seen only in musth. Most males (94%) were more often seen foraging than in musth. Musth-only males were least likely to be seen across all 6 years of the study, whereas those seen in both states were most likely to be seen across years (Table 1). Those seen only foraging were largely split among those seen only in 1 year (38.4%) and those seen in all six (11.1%). There was a significant association between age and strategy, with younger males more likely to be seen only foraging, whereas prime-aged and older males were more likely to be seen in musth-only or both states (Fisher’s exact test p < 0.0001). The propensity to be seen in musth peaked in the 31–40 age class, declining thereafter (Fig. 2A). For n = 12 males whose full musth period was observed, musth duration lengthened with age with a median duration of 24.5 days for ages 21–30, 32.5 days for ages 31–40, and 45 days for those > 40 (Fig. 2B). Males seen in both states were more frequently seen in the early-phase whereas musth-only males were seen slightly more frequently in the peak- and late-phases (Fig. S3 and Fig. 2C).

**Table 1:** Number of mature males (≥ 21 years) using each strategy across years. N = 216 (musth-only (m) = 23 (11%), foraging-only (f) = 99 (45%), foraging and musth (f + m) = 94 (44%)). Musth-only males are the least reliably seen across years whereas those seen in both states are most often seen across years.

Frq (yr)	Musth-only	%	Foraging-only	%	Foraging and musth	%
6	–	–	11	11.1	29	30.9
5	–	–	9	9.1	22	23.4
4	2	8.7	9	9.1	14	14.9
3	1	4.3	14	14.1	9	9.6
2	3	13	18	18.2	15	16
1	17	73.9	38	38.4	5	5.3

There were 2732 male-weeks of sightings across the 216 mature males where 343 (12.5%) included musth and 2394 included only foraging. Among the subset of 31 putative residents, the total number of male-weeks observed was 1308, split into 93 (0.71%) musth and 1215 foraging. The proportion of musth male-weeks in this subset was significantly less than that of the full sample (exact binomial test, p < 0.0001), but there was no significant difference in the age structure (*X*^2^ = 0.57569, df = 2, p = 0.749), therefore it was not age-driven.

**Figure 3:**
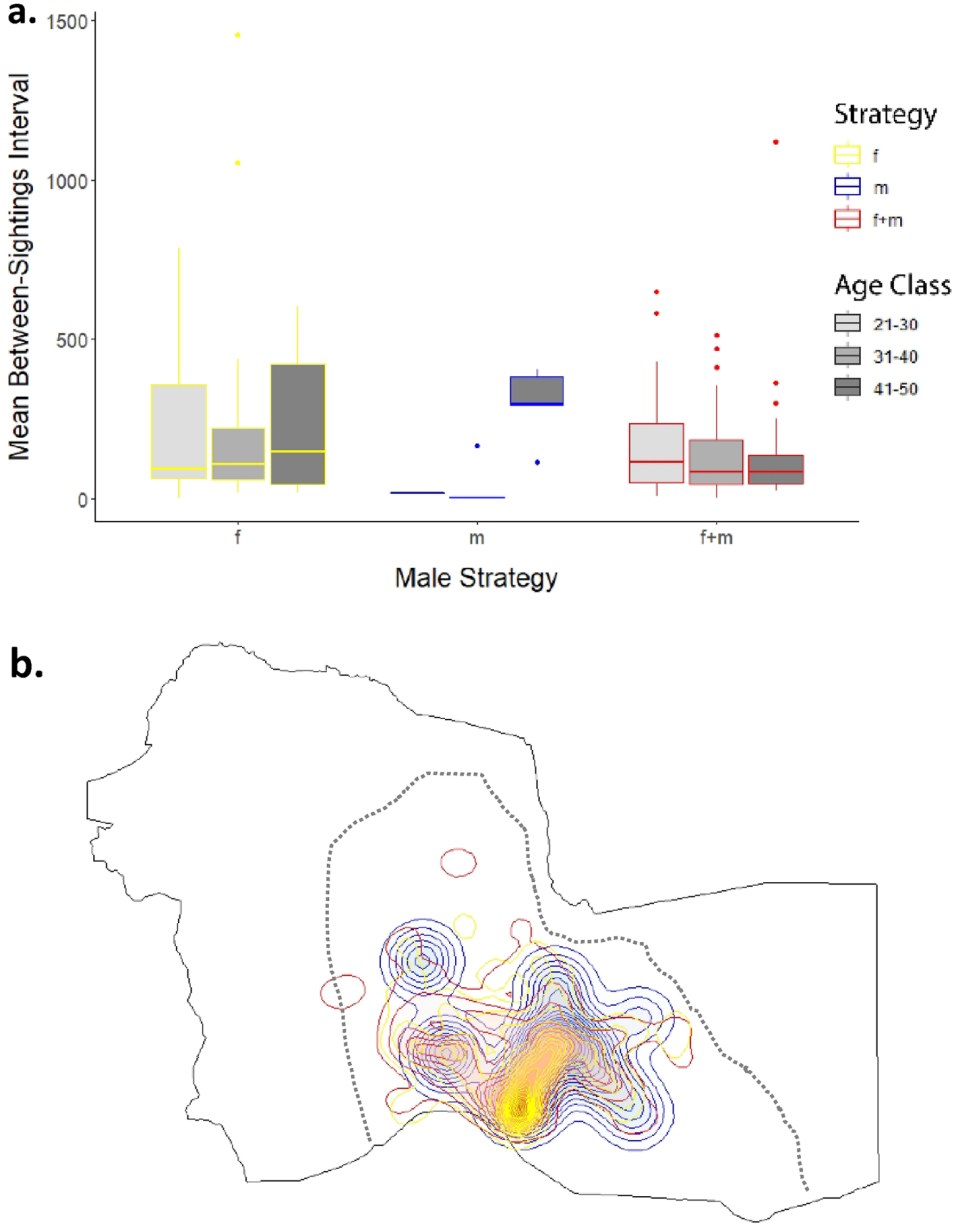
Male PA-use by age and strategy. (**a**) Average length of between-sightings interval (BSI) by male age and strategy. Males employing the foraging-only strategy tend to have longer BSIs on average than those employing other strategies, while age is not overall an important influence. However, there was an interaction of age and strategy, with BSIs of musth-only males being significantly shorter than those with other strategies. This was driven by males aged 21–40, while BSIs of males in the 41–50 age class were significantly longer (see Table 3). (**b**) Space use of males following each of the different strategies, indicated by the same color as (**a**). Males exhibiting different strategies show no visible spatial differentiation within our central observation area. Observation area is defined by the dotted line; lack of data outside this is simply due to sampling, not absence of elephants.

The between-sightings-interval (BSI), measured in days, provides a measure of time that subjects are potentially away from the study area (Fig. S4, see “Methods”). The sample size for the GLMM was reduced from 216 to 176 after removing the oldest age class (n = 2) and males seen only once, for whom BSI was undefined (n = 38). There were three candidate models to test the relationship between BSI, male age class and motivational state (Table S1). The model including an interaction between age class and state performed best (ANOVA p < 0.0001; Table S1), with results in Table 2. Males following a foraging-only or mixed strategy of PA-use had similar BSIs whereas those following a musth-only strategy were split: the younger age classes showed the lowest BSIs while the oldest age class showed the highest of any category (Fig. 3A). Males employing different strategies did not segregate spatially within the observation area (Fig. 3B).

**Table 2:** Male BSI: model output from highest performing generalized linear model. Males following the musth-only (m) strategy showed significantly lower BSI values than males using the foraging-only (f) strategy or those doing both (f + m). Male strategy and age class interacted significantly for musth-only (m) at the highest age class, with higher BSI values than other categories.

Fixed effect	Estimate	SE	t-value	p-value
Intercept	4.452123	0.529418	8.409	< 2e−16*
Musth only	− 2.103830	0.912723	− 2.305	0.0212*
Foraging and musth	− 0.006498	0.361235	− 0.018	0.9856
31–40	0.164912	0.296016	0.557	0.5775
41–50	0.559655	0.513164	1.091	0.2755
Musth only: 31–40	− 0.657094	1.027345	− 0.640	0.5224
Foraging and musth: 31–40	− 0.550566	0.453086	− 1.215	0.2243
Musth only: 41–50	2.517846	1.088762	2.313	0.0207*
Foraging and musth: 41–50	− 0.865966	0.618111	− 1.401	0.1612

### Female PA-use

**Figure 4:**
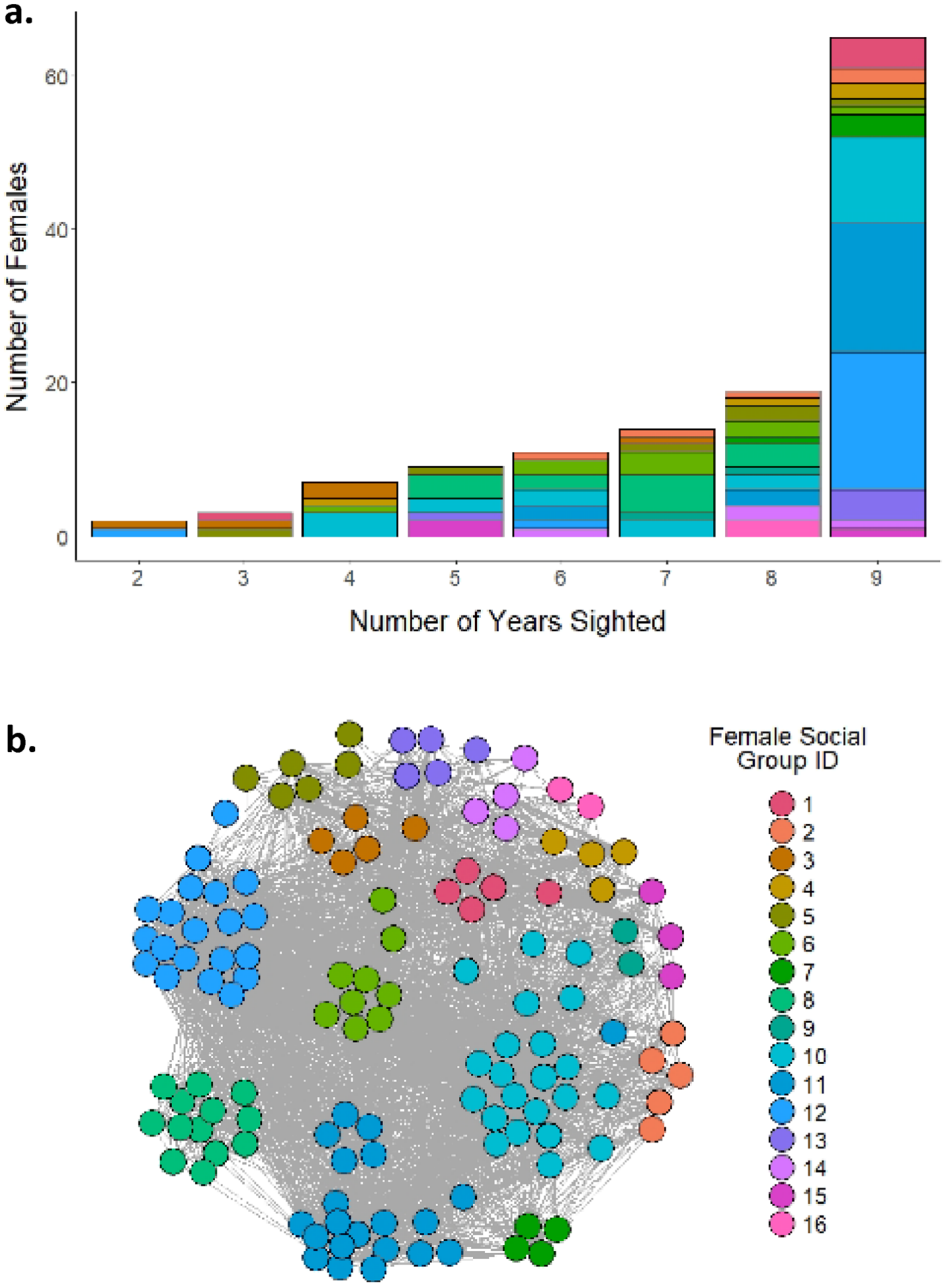
Sightings and social affiliations of adult females (n = 130). (**a**) Individuals were required to be seen in both of the first years of the study in order to be included in our sample but half (65 individuals) were seen across all nine. Colors indicate community assignment; individuals from the same community differ in how frequently they were seen. (**b**) Community size and structure.

**Figure 5:**
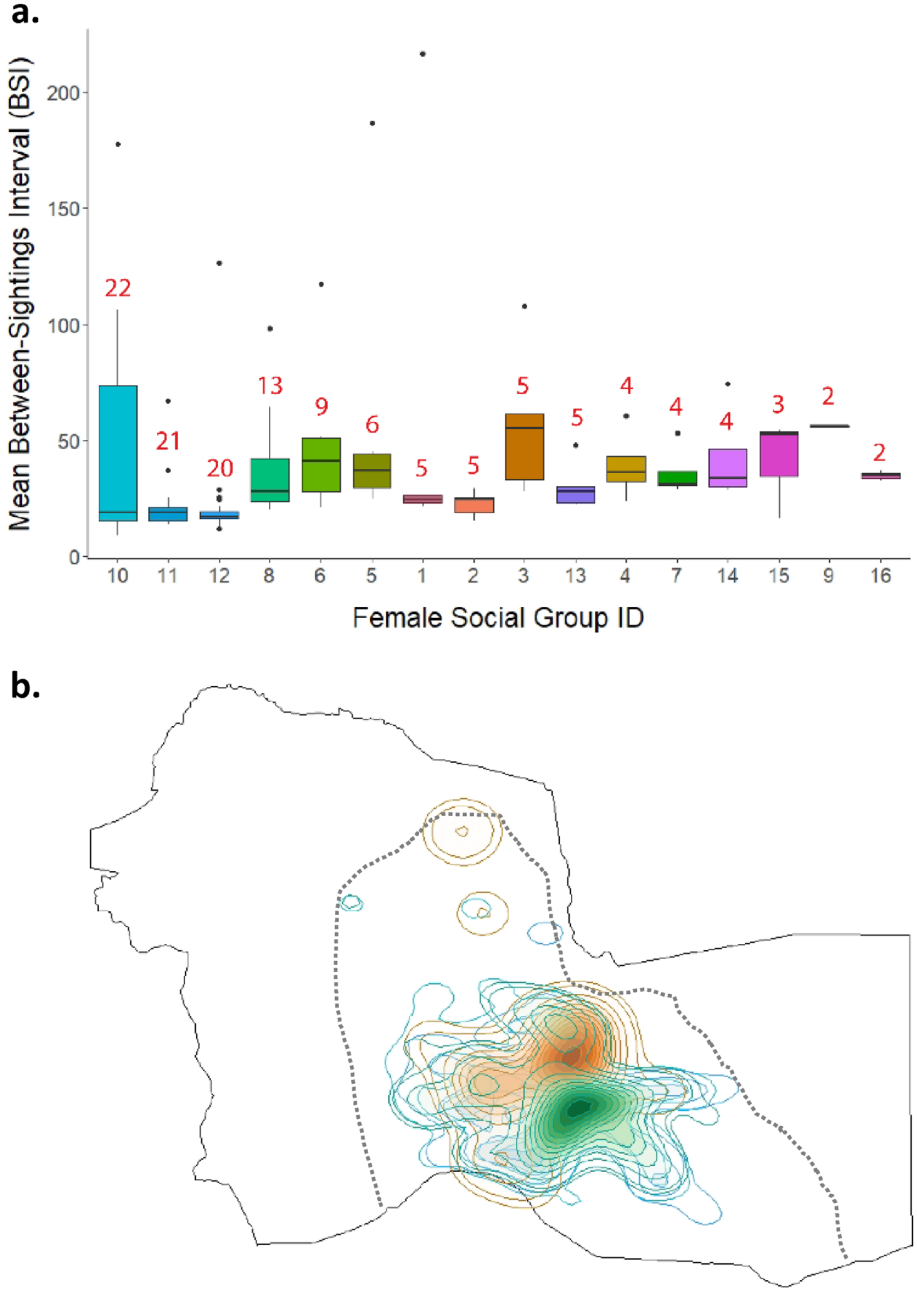
Female BSI and space use. (**a**) Mean between-sightings-interval (BSI) for adult females when individuals are grouped by social assignment. Numbers along the x-axis correspond to community identity (Fig. 4) and are ordered by community size from largest (left, 22 individuals) to smallest (right, 2 individuals). (**b**) Space use for 2 communities with the highest (orange and green, communities 3 and 9) and lowest median BSI (turquoise and blue, communities 10 and 12), illustrate high spatial overlap between putative residents and non-residents within the observation area, defined by the dotted line.

There were 300 females identified, of which 230 were adults. Of these, 137 were seen consistently throughout the first 2 years of the study, but seven females known to have died later and were removed from the dataset (n = 130 remaining). Half of these females were re-sighted in each year, while 0.2% percent were not seen again and may represent additional mortality or dispersal events (Fig. 4A). Using pairwise association indices (Simple Ratio Index or SRI^46^), we assembled a female social network for each year of the study to represent long-term relationships over the annual cycle rather than short-term fission–fusion dynamics. We used a clustering algorithm to detect social communities based on network structure within the first 2 years of the study (see “Methods”^47^), and identified communities that persisted across years (see “Methods”^48^). Social community size ranged from 2 to 22 individuals (Fig. 4B). The largest social communities had the lowest median BSI values (Fig. 5A), suggesting that larger communities have more residential members than smaller communities, but this cannot be statistically evaluated as individuals within communities are non-independent (see below) and community size is a variable with only 16 values. Communities exhibited slight differentiation in observed core areas but considerable spatial overlap overall, given our limited sampling area (Fig. 5B).

We used a multiple regression quadratic assignment procedure (MRQAP) to assess how social associations (using the simple ratio index, or SRI^49^) affect similarity in female PA-use (Pearson’s pairwise correlation in BSI; see “Methods”). This model showed a significant relationship (p < 0.001, Table 3) between the association matrix and the BSI similarity matrix after accounting for gross spatial overlap measured in terms of the Euclidean distance between the range centroids for each individual. This shows that social associations predict BSI similarity, which as a proxy for PA-use, suggests that females and their close social partners have similar temporal use of the PA. However, the effect was weak (R^2^ = 0.01), likely due to the fission–fusion process and high individual variation in long-term sightings as observed in Fig. 4A.

**Table 3:** Female BSI: model output from multiple regression quadratic assignment procedure (MRQAP). Pairwise female BSI was significantly correlated with the simple ratio index after controlling for the Euclidean distance matrix.

Independent variable	Estimate	Two-tailed p-value
Intercept	0.86	< 0.001
Association matrix	0.43	< 0.001
Spatial matrix	− 0.46	0.47

Adjusted R^2^ = 0.01, Residual SE = 0.18, df = 8382.

## Discussion

The scale of space-use by large mammals is mismatched with the scale of PAs, a challenge that is not limited to migratory species or large carnivores^50–52^. This is especially true in Asia, where PAs are orders of magnitude smaller than counterparts on other continents^19,53^. Because space-use is dictated by life history, we examined how these attributes might affect PA-use among Asian elephants. We found that PA-use strategies vary among individuals of the same sex and even social associates within a given population. Observations also suggest nearly all adult males and at least half of adult females (i.e. ~ 3/4 of the population) may not be long-term residents. We consider these observations in more detail and the issues they raise for the reliance on PAs for accommodating elephants and other wide-ranging taxa within highly-modified landscapes.

Among males, there was a significant trend for younger males to use it exclusively for foraging. Males following this strategy included a residential minority, but close to 90% disperse at some point (Table 1). Males using the PA during both states were more likely to be seen across years. Two sets of results indicate that males who are initially foraging in the park appear to be leaving once they enter musth. Males following a mixed strategy were more likely to be seen in earlier musth stages than those following a musth-only strategy (Fig. 2B), and residential males use the area significantly more for foraging than the male population as a whole. As observed in other studies of wild and captive elephants^35,54^, it is likely these males gradually decrease their food intake as musth progresses and range more extensively before returning to the park, if they do so at all. On the other hand, musth-only males mostly enter the study area during the peak-phase and many stay through the late-phase, but are not observed feeding, presumably because they leave the study area before they re-commence foraging. Repeated sightings across years were rarest for this group (Table 1), similar to observations from India^31^. Interestingly, there was a shift in strategy with age (Fig. 3A). Most musth-only males aged 20–40 were highly mobile and thus seen often when present (at which time they had low BSIs), but did not return annually (undefined BSIs). This indicates that even fully sexually mature males engage in decades of exploration during their musth periods, without returning to any particular area on a regular basis. In contrast, males > 40 years appear to have settled into a more regular cycle of visitation during musth, evidencing significantly longer (inter-annual) BSIs relative to other classes. This recurrent ranging pattern may result from an extended process of male-male contests, eventually leading to temporal-partitioning with other similarly-aged males such that they are not in direct competition during their musth periods. Studies of African savannah elephants indicate that older males may suppress the musth of younger males and severely limit their access to oestrus females^55–58^. The duration of musth is likewise observed to increase with age in African savannah elephants^56,59^. The oldest males (> 40), having established reproductive dominance at a particular time of year, may therefore return predictably to the same area on an annual basis. However, we find no evidence of spatial partitioning suggestive of competitive exclusion among bulls following different strategies.

The varying strategies employed by males in this population demonstrates that remaining in a limited area during both motivational states is not a viable option despite the PA’s relative safety, availability of forage, and presence of many females. The median birth interval for females in this population is 6 years^67^, and oestrus on average may last just 4 days^32^. Quite possibly, the reason that elephants evolved musth in the first place is related to a foraging-reproduction trade-off similar to what is faced by cervids such as white-tailed deer^60^. Since scramble competition for scarce oestrus females forces males to roam widely, they likely move away from preferred foraging grounds. For those who preferentially forage inside the PA, this entails leaving it, whereas for those who preferentially forage outside, it entails entering it. However, males’ ranging needs present potential risks to people outside the PAs. Anecdotally, three of the identified bulls were removed from the vicinity of PA and placed in a holding facility by authorities, as they were viewed as posing a risk to humans. At least one was implicated in three human fatalities near the study area. All three were rarely seen in the PA during the study period and all exhibited signs of musth when captured (SdS personal observations). It is possible that these fatal encounters resulted from males ranging into unfamiliar areas during musth-induced range expansion, whereas those who are more familiar with the area are less likely to pose a risk (see also^61^). Nevertheless, there have been fewer than five such incidents during the course of this study, and given that males in this population seem largely transient, the rarity of such encounters is remarkable. If the captured males were indeed accountable, campaigns of public awareness focused on how to recognize and avoid musth males, together with communally-managed notification systems may further reduce the chances of a deadly encounter with males that otherwise pose no threat.

We expected that female PA-use would be related to social relationships, because associations are defined in terms of spatiotemporal co-occurrence. Accordingly, SRI was significantly correlated with BSI for pairs of individuals. However, this relationship was very weak. This is because the fission–fusion process introduces variation among individuals within the same putative community. We found that members of the same community were not necessarily seen at similar frequencies across years, which suggests that individuals belonging to the same social community do not necessarily disperse together as a unit or associate very closely over the entire 9-year period. Earlier studies of this population found that on average, the number of companions an individual had was negatively correlated with the strength of her ties^40^ and that the fission–fusion process undermines enforcement of dominance hierarchies^44^. We also found a tendency for individuals from larger communities to have lower BSIs, and given that many were seen across multiple years, these individuals were likely to be more residential to the observation area. The smaller communities with less residential individuals (i.e. longer BSIs), may be part of social units with other members that are rarely in the study area and therefore not seen. Thus, PA-use reflects some combination of individual decision-making and socially-associated movements. Community members may split up owing to local competition and constraints on group size^41^. When individuals compete, dominance hierarchies typically function to mitigate conflicts. Strong hierarchies, such as those observed in African savannah elephants^62,63^, can mediate priority of access to resources, or even safe zones that are more central to PAs^64^. Because Asian elephants in this population do not exhibit dominance hierarchies, spatiotemporal avoidance may instead buffer against conflicts. Thus, individuals may move to less accessible areas within the PA, or outside it entirely (observed anecdotally), *without* their social companions. This individual-level avoidance rather than hierarchical exclusion is further reflected in the lack of clear spatial segregation by social communities in this population (Fig. 5).

Asian elephant populations have been greatly impacted by human-induced land cover changes that reduce both the extent and connectivity of habitat^17^. Such conversions often replace rangelands with fenced or otherwise restricted cropland^25,65,66^. Human proximity decreases foraging efficiency, as it necessitates avoidance of human activities within remaining rangelands and habitat edges^26,66^. Failure to do so can lead to negative interactions with people^25,67,68^. Rathnayake et al.^25^ document that 98% of conflict incidents with elephant in Sri Lanka take place within 1 km of a recent land-use conversion. While setting aside PAs may be perceived to stem these issues, studies elsewhere in South Asia suggest suitable habitat is being lost even inside protected areas^69,70^. Within this context, our findings underscore that elephants of both sexes regularly seen *inside* a highly-visited and economically valuable National Park are likely also reliant resources outside it. Because females are less transient than males, lack of access to adequate habitat potentially contributes to the extremely slow reproductive rates documented in this population, which can result in long-term population decline^67,71^. On the other hand, the ranging requirements of males create risk of conflict as land-uses change, as previously discussed. For wildlife managers and policy makers, safeguarding elephant populations entails managing relationships with communities to ensure that adequate resource access can be safely maintained. As a case in point, the Dahaiyagala sanctuary (a narrow “corridor” extending northward from the National Park) has been repeatedly encroached, resulting in land disputes^72^, but serves as a vital link to forage and resources beyond the PA that are themselves threatened. While these external, largely unprotected (or mixed-use) landscapes may not be seen as being especially valuable in terms of either economic value or biodiversity, their role in maintaining the demographic health of elephant populations and reducing conflict potential remains under-appreciated.

A study in Malaysia suggested that agricultural landscapes might be prime habitats for elephants^73^, while studies in Indonesia and Borneo document the propensity of elephants to exploit “degraded” areas associated with forest edges, often outside PAs^74,75^. Although primary forests are often prioritized for biodiversity conservation^76^, elephants may prefer to forage in secondary and regenerating landscapes. But we must be extremely careful in interpreting and generalizing from these studies. First, PAs globally are biased towards steeper, higher terrain^7^. In Southeast Asia, PAs often consist of rugged terrain, boxing in elephants when lowland valleys are rapidly converted to other land uses^66^. Use of “edge” habitat and agricultural areas may reflect the lack of adequate, low-risk, preferred lowland forest habitat. Much forest has already been lost throughout Asia through conversion to intensive agriculture, contrasting with pre-colonial systems of management^77^. In Sri Lanka, very little of the lowland rainforest remains, having been settled and cultivated since the 1800s. Thus many populations remaining today may have no choice but to use suboptimal human-dominated landscapes, despite associated risks and costs^78,79^.

It is interesting to note that many PAs in South Asia run counter to the trend, being frequently located around rivers and other water bodies, largely to protect their catchment zones, but which also seasonally host significant populations of elephants and other wildlife. Their attraction to these PAs likely has to do with the presence of both water and monsoon-mediated forage (e.g. floodplains). However, the water resources of these PAs may be increasingly expected to meet agricultural needs, especially in the face of climate change, but may be counterproductive, as it raises conflict potential when elephants and other wildlife are displaced from within PAs. In a separate example, the approximately 1700 year-old Minneriya Reservoir in central Sri Lanka, now also centered within a National Park, famously hosted elephants by the hundreds during dry seasons. It was kept in inundated in 2021, during which such a gathering did not occur; elephant incursions on croplands in surrounding communities concurrently increased^80^. Our observation that a substantial fraction of the population is non-residential reiterates the importance of maintaining the functionality of PAs *as part of* wider connected landscapes as different segments of the population vary their space use according to their specific needs. Individual-based studies of how other wildlife actually use PAs as well as in human-dominated landscapes^24,81^, though logistically daunting, would further illuminate how strategies vary in response to anthropogenic changes.

## Methods

### Study area and population

We observed wild Asian elephants inside Udawalawe National Park in southern Sri Lanka. The park is approximately 308 km^2^ and contains two reservoirs resulting from the damming of the Walawe river and a tributary, as well as several smaller water sources, situated in a seasonally dry/deciduous scrubland. The PA is encircled by electric fences, however intentional openings as well as breakages allow the movement of wildlife in and out. The superpopulation of elephants using the habitat is estimated to be 804–1160 individuals with a sex ratio of 1.18 in favor of females, with a high degree of seasonal turnover such that only approximately one third to one half the population is within the PA at any given time^45^. Males were observed from 2010 to 2015 and females were observed from 2007 to 2015. Observations were made by vehicle between 0600 to 1830 hours on tracks driven along a randomly determined route^40^. Individuals were identified through photographic cataloguing primarily using features of the ears^82,83^. The location of each sighting (which could include one or more individuals) was recorded using a hand-held Garmin GPS. The identities of all known individuals, the number of unidentified individuals, and the number of individuals in non-adult age classes was also recorded^40^.

### Quantifying PA-use

We quantified the temporal structure of individual PA-use by calculating the number of days that elapsed between consecutive sightings of a given individual, which we termed the “between-sightings interval” (BSI). It provides a measure of how often an individual is in the study area, while accounting for the impossibility of knowing exactly when they enter and exit the PA. A shorter average BSI can indicate that an individual is remaining close to the study area, while a longer BSI allows for the possibility that it ranges further afield. It may be thought of as complementary to residence time, which cannot be defined from sightings alone. To avoid introducing intervals of artificial length, the average BSI for any individual was only calculated over the first and the last sighting for each individual. This necessarily omitted possible inter-annual variation for individuals observed only within a single year (Fig. S1).

### Defining age classes and strategies for males

Males were assigned into four coarse age classes based on height and the development of secondary sexual features: 21–30, 31–40, 41–50, 51–60. Physiologically, individuals in all classes are capable of being reproductively active and exhibiting musth^32,84^. Younger sub-adult males and males that transitioned into maturity were excluded from statistical analyses. An individual male’s strategy was defined as “foraging-only” (f), if he was *never* seen exhibiting any signs of musth across the study period, “musth-only” (m), if he was *only* seen in the musth condition across the study period (i.e. never observed foraging), and “foraging and musth” (f + m), if he was seen in *either* state at any time during the study (which could include foraging either while in musth, which was rare, or more commonly nonmusth). For males observed in musth, we also recorded the stage of musth as early, peak, or late based on physical appearance (Fig. S2; see also^35,39^). For males whose musth period was observed from beginning to end with no intervening non-musth sightings, the duration of the musth period was calculated in days elapsed between the first and last musth sighting.

### Defining social communities for females

To quantify adult female social relationships, we created a group-by-individual matrix from all observations of adult females and calves between 2007–2015. The full dataset was then filtered to include only adult females that were present in the first 2 years of the study to allow for the possibility that they were available for observation over the full duration of the study (n = 130). Next, we constructed an association matrix using simple ratio index (SRI) as edge weights in the R package *asnipe*^49,85^. SRI is a pairwise index of association that describes the proportion of observations where two individuals were seen together out of all possible observations^46,86^. An association matrix and corresponding social network were constructed for each of the first 2 years of the study separately. We then used a Louvain clustering algorithm (package *igraph*^47^) to detect communities within each of the social networks, and used a dynamic community detection algorithm^48^ to identify whether social communities were the same between the 2 years. This algorithm used a reciprocal majority method, i.e., any network clusters that had more than half of the same members between subsequent years were considered the same social community. Each individual was therefore assigned to a particular community based on the first 2 years of observation, which was then nominally retained for subsequent years.

### Analysis

Statistical analyses were conducted in Microsoft Excel™ and R (R development core team 2019). For individual males, we calculated the proportion of sightings for musth vs. non-musth in weeks as opposed to days because sampling did not take place on a daily basis. The unit of measurement is therefore referred to as “male-weeks.” Sightings were aggregated by week for each individual and scored as 0 if not seen within that week, 1 if seen only foraging, and 2 if seen in musth at any time during that week. To test whether possible residents (defined as BSI < 90 days and seen across multiple years) were observed foraging more or less than the overall population, we compared the proportion of male-weeks in musth to that of the whole population using an exact binomial test (two-tailed).

To test whether male BSI was influenced by age class or musth state, we constructed a series of generalized linear mixed models (GLMM) using a gaussian logit link function in the R package *lme4*^87^. For each model, BSI was the dependent variable and the random effects were individual ID, month, and year. The possible covariates, motivational state and age class, were tested for correlation using a Fisher’s Exact Test to determine how to structure models, i.e., if age class and state should be included as covariates in models. We evaluated and selected the best fitting model using an analysis of variance test (package *stats*, R Core Team 2021).

To test whether social associations explain similarity in female PA-use, we used a multiple regression quadratic assignment procedure (MRQAP^88^; R package *asnipe*). We used three matrices of pairwise metrics (one for the dependent variable and two for each independent variable) to discern the relationship between social association and similarity in BSI while accounting for spatial autocorrelation. The dependent variable was a matrix consisting of the pairwise Pearson’s correlation coefficient for the average BSI of each female with every other female across all 8 years. The first independent variable was the pairwise SRI association matrix, representing the strength of association between individuals. The second independent variable accounted for spatial autocorrelation by calculating a pairwise index of spatial use (i.e., the spatial matrix). This was done by calculating a centroid for each female using GPS coordinates from all sightings of that individual. We then calculated Euclidean distances among the centroids of each pair of individuals to represent similarity in their space use. The MRQAP model tests whether the dependent matrix (pairwise correlation in average BSI) is explained by the independent matrix (pairwise association index) while controlling for the non-independence of the covariate matrix (pairwise Euclidean distance between centroids).

## Supplementary Information

Supplementary Information.

## Data Availability

Data will be made available in a repository such as Dryad upon final acceptance. For review purposes, the raw data files can be accessed via the temporary link: https://drive.google.com/drive/folders/1gWniY3useFT03XOIZ06tnFu3AH_yCqJt?usp=sharing.
