# Supplementary material for: Strategies of protected area use by Asian elephants in relation to motivational state and social affiliations
Source: Sci Rep. 2022 Nov 2;12:18490. doi: 10.1038/s41598-022-22989-1 (PMC9630427; doi:10.1038/s41598-022-22989-1)
Supplement: Supplementary file 1 — Supplementary Information. [file 41598_2022_22989_MOESM1_ESM.pdf]

Strategies of Protected Area Use by Asian Elephants In Relation to Motivational State and Social Affiliations

Supplementary Material

Anastasia E. Madsen[1*], Christin Minge[2,3*], T.V. Pushpakumara[4], U. Sameera Weerathunga[4], U. K. Padmalal[5], Devaka K. Weerakoon[6], Shermin de Silva[2,4,7†]

[1]University of Nebraska-Lincoln, Lincoln NE, USA
[2]Trunks & Leaves Inc., Newtonville, MA USA
[3]Friedrich-Schiller University, Institute of Ecology and Evolution, Jena, Germany
[4]EFECT, 215 A 3/7 Park Road, Colombo 5 Sri Lanka
[5]Open University of Sri Lanka, Colombo, Sri Lanka
[6]University of Colombo, Colombo, Sri Lanka
[7]University of California, San Diego, La Jolla CA 92093 USA
*Equal contributions
[†]Corresponding author: shermin@elephantresearch.net

Keywords: *Elephas maximus*, protected areas, space use, musth, social networks

Figure S1 - Cumulative discovery curve for males, N = 379.

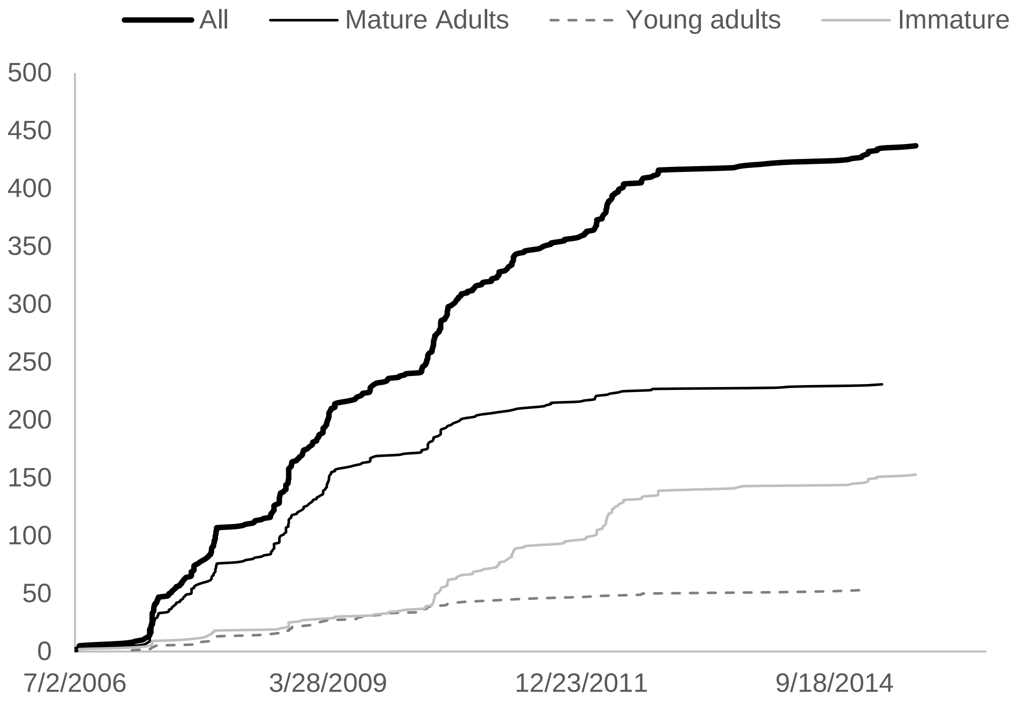

Figure S2 - Sightings distributions, N= 216. (a) Aggregate across all individuals. **(b)** By age-class.

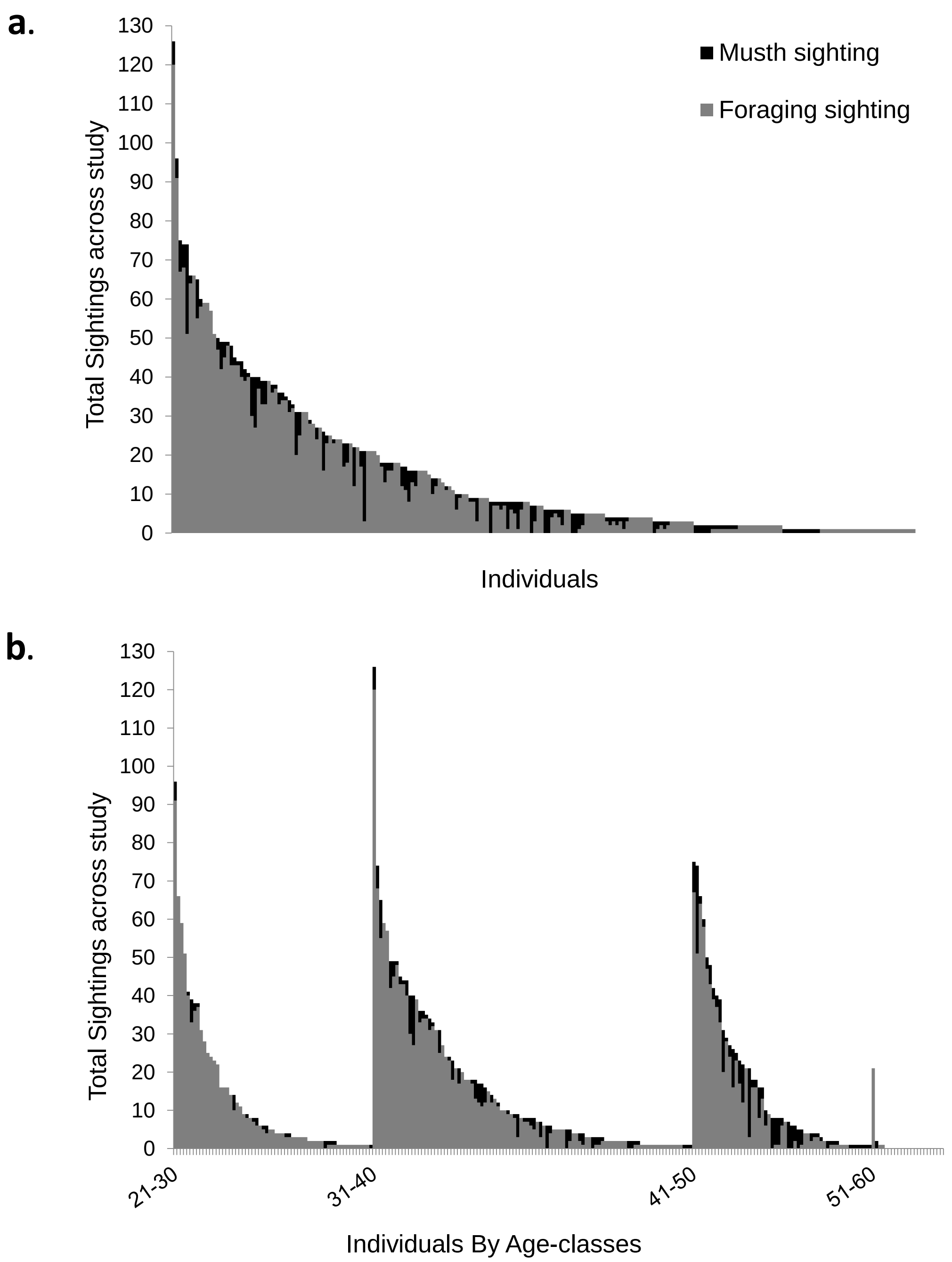

Figure S3 – Musth stages. (a) Early-musth is characterized by light temporal secretions and urine dribbling. **(b)** Peak-musth is identified by distinct wet patches on the temples, often with reddish coloration, and constant urine dribbling staining the hind legs. **(c)** Late-musth is identified by reduced temporal secretions and urine dribbling. It is distinguished from early-musth by the presence of dried secretions on the temples and dried urine residue on the sheath of the penis and hind legs.

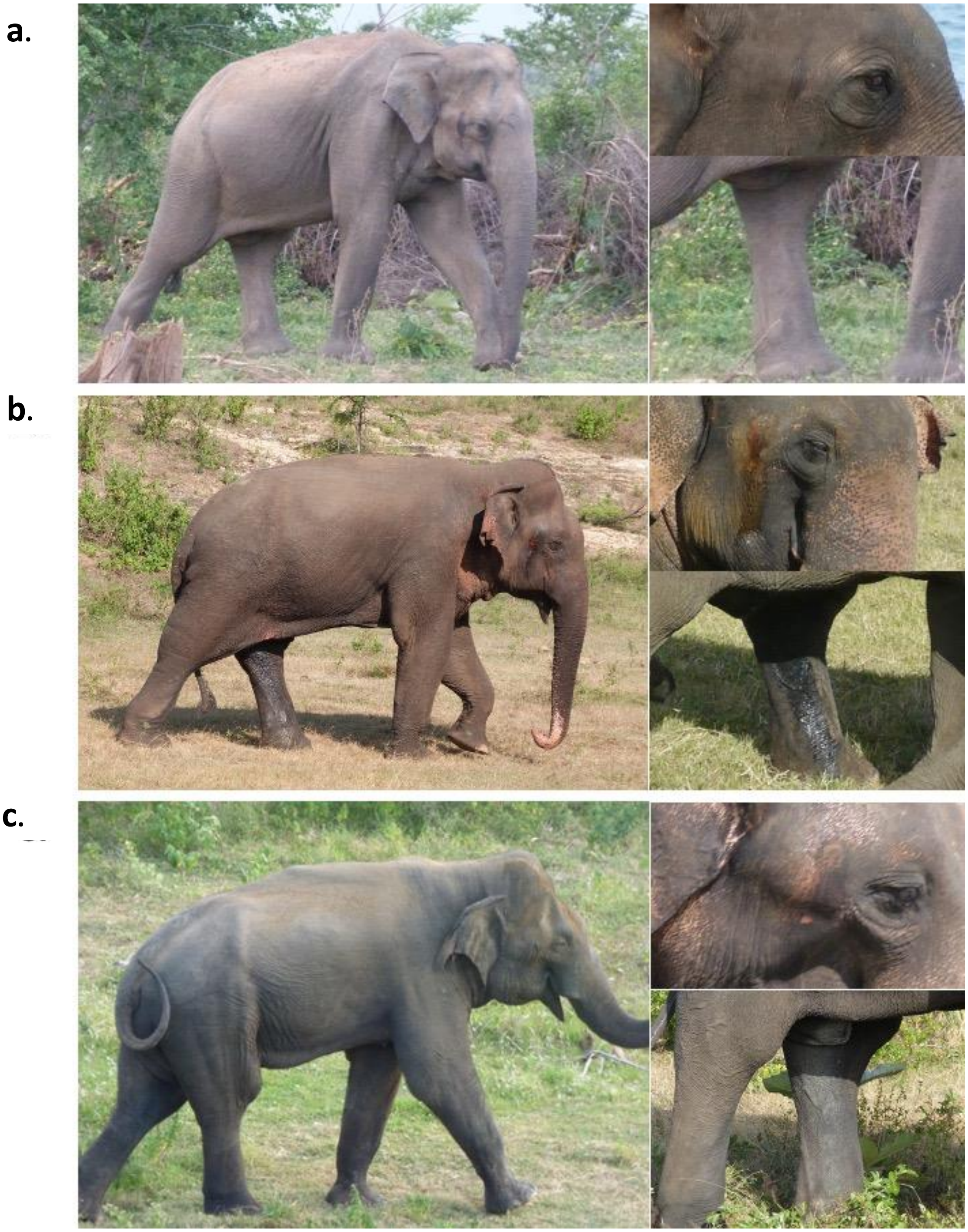

Figure S4 – Schematic representation of the Between-Sightings Intervals (BSI). BSI is defined as the number of days passed since the previous sightings of an individual, and was calculated for each individual starting with its first sighting, and ending with its last sighting. A snapshot of 25 days within the study period with 3 hypothetical individuals is represented. The horizontal light gray bands represent the continued duration of the study period (25 days here), the darker gray and black bars represent discrete daily sightings (see legend below), and white bands represent days between sightings (numbered). For each elephant, a mean BSI is calculated from the days passed between sightings, as tabulated to the lower right of each band.

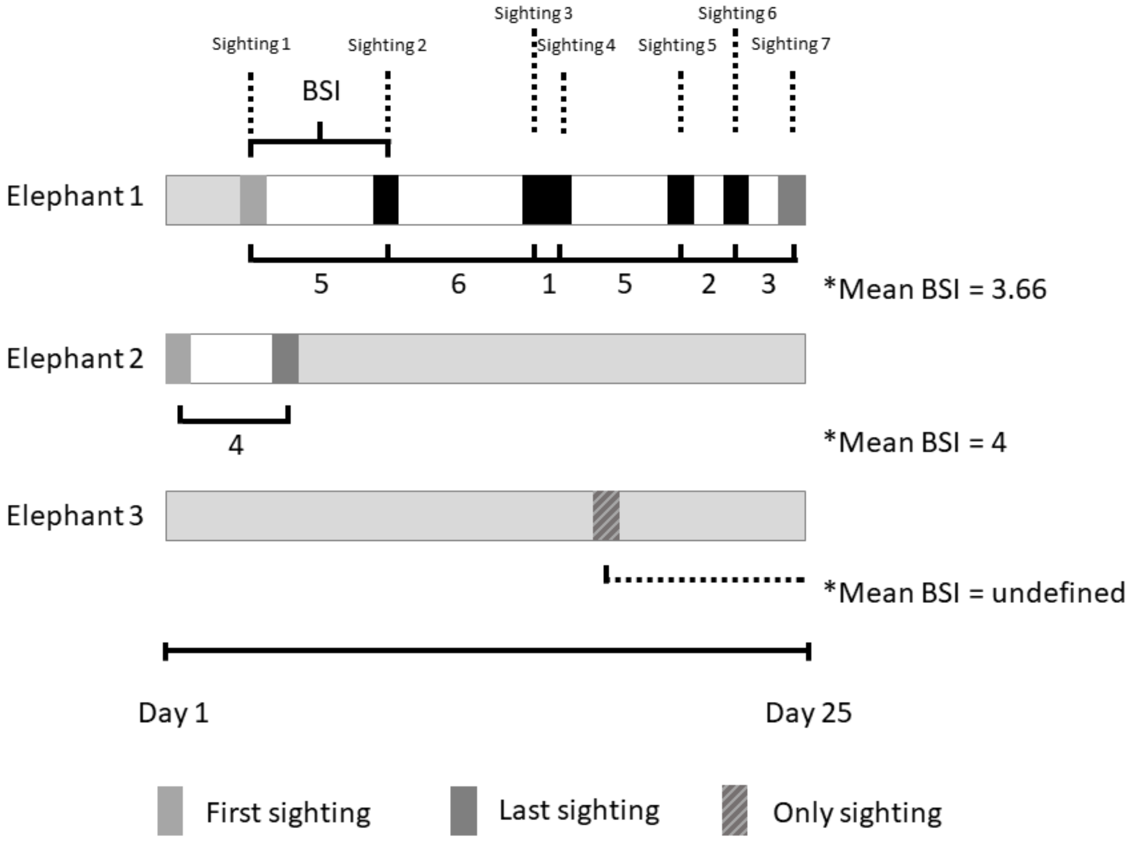

Table S1 – Generalized linear mixed model selection for male between-sightings-interval (n=176 males) Possible covariates were strategy (musth-only, foraging-only, or foraging and musth). Three candidate models and a null model were included for comparison during model selection.

Model	K	AIC	ΔAIC	log Likelihood	Deviance	p-value
*BSI ~ strategy * age class +(1|ID)+(1|Month)+(1|Year)	13	38062	0	-19018	38036	$8.03*10^{-5}$
BSI ~ strategy +(1|ID)+(1|Month)+(1|Year)	7	38073	11	-19029	38059	0.023
†BSI ~ (1|ID)+(1|Month)+(1|Year)	5	38076	14	-19033	38066	-
BSI ~ age class+(1|ID)+(1|Month)+(1|Year)	7	38079	17	-19032	38065	-

*selected model, †null model
